# Association between the LINC00673 rs11655237 C> T polymorphisms with cancer risk in the Chinese population: A meta-analysis

**DOI:** 10.1097/MD.0000000000030353

**Published:** 2022-09-16

**Authors:** Hongyu Zhang, Baixiu Wu, Ka Liang, Liuhua Ke, Xingxuan Ma, Changliu Luo, You He

**Affiliations:** a Department of Clinical Laboratory, The Third Affiliated Hospital of Guangxi University of Chinese Medicine, Liuzhou Traditional Chinese Medical Hospital, The Third Clinical Faculty of Guangxi University of Chinese Medicine, Liuzhou, Guangxi, China; b Department of Gynecology, the Fourth Affiliated Hospital of Guangxi Medical University, Liuzhou Worker’s Hospital, Liuzhou, Guangxi, China.

**Keywords:** cancer risk, LINC00673, meta-analysis, polymorphisms

## Abstract

The present study aimed to conduct a meta-analysis of previously published studies in order to clarify the association of long noncoding RNA (lncRNAs) LINC00673 rs11655237 C> T polymorphism with cancer risk.

**Design:**

Systematic review and meta-analysis.

**Setting:**

Electronic databases of PubMed, EMBASE, Web of Science, Cochrane Library, Chinese National Knowledge Infrastructure, and Wanfang Database were used to search relevant studies. Studies published up to October 20, 2019 were included. The included studies were assessed in the following genetic model: allelic model, homozygote model, Heterozygote model, dominant model, recessive model. Data syntheses were conducted using STATA 12.0.

**Participants:**

Participants with various types cancers were included.

**Primary and secondary outcome measures:**

Odds ratio (ORs) and 95% confidence interval (CIs) were calculated to assess the risk of tumor.

**Results:**

Seven articles including 7 case-control studies, 7423 cases and 11,049 controls were adopted for meta-analysis. Our result demonstrated that LINC00673 rs11655237 C> T was related to the cancer among all model including allelic model (T vs C: pooled OR = 1.24, 95% CI = 1.16–1.41, *P* < .001), homozygous model (TT vs CC: pooled OR=1.54, 95% CI = 1.36–1.76, *P* < .001), heterozygous model (CT vs CC: pooled OR=1.24, 95% CI = 1.16–1.32, *P* < .001), dominant model (CT + TT vs CC: pooled OR=1.28, 95% CI = 1.20–1.36, *P* < .001) and recessive model (TT vs CT+ CC: pooled OR=1.42, 95% CI = 1.25–1.61, *P* < .001). Subgroup analysis also demonstrated that polymorphisms at this site also increased the risk of neuroblastoma.

**Conclusions:**

Our results find that rs11655237 contributed to occurrence of cancer in all models in Chinese population.

**Key Points**

• This meta-analysis showed that the LINC00673 rs11655237 C> T polymorphism can increase cancer susceptibility.
• In our meta-analysis, only the relationship between the polymorphism of LINC00673 rs11655237 C> T and the susceptibility of tumor was studied. Other sites of the gene and the interaction of genes with the external environment were not analyzed.
• Small sample size may be the biggest research defect, which may affect the result and lead to incorrect conclusions.
• The subjects included in the study are all Chinese, so racial bias is inevitable and affects the outcome.

## 1. Introduction

Cancer is the second leading cause of death in the world, with about one-sixth of deaths caused by cancer, and about 70% of cancer deaths occur in low- and middle-income countries.^[1]^ Lung cancer is the most common cause of cancer-related deaths worldwide, accounting for 19% of all cancer-related deaths.^[2]^ Smoking, drinking, unhealthy diet and lack of exercise are recognized as major cancer risk factors worldwide.^[3]^ Of course, some carcinogenic infections, including Helicobacter pylori, human papillomavirus, hepatitis B virus, and hepatitis C virus, can greatly increase the risk of cancer. If cancer can be detected and diagnosed early, it can not only reduce cancer mortality, but also improve survival rate and greatly improve the quality of life of cancer patients.

Non-coding RNAs (ncRNAs) are RNAs that do not encode proteins. These RNAs can be transcribed from the genome, but they are not translated into proteins, and they can perform their biological functions at the RNA level. ncRNAs can be classified in length, and lncRNAs are >200 nt in length. The number of lncRNAs has an absolute advantage in ncRNAs. Compared with the encoded protein gene, it has higher tissue-organ specificity and can be found in blood, urine, tumor tissue, or some other tissues or body fluids, and has the potential to become Biomarkers for cancer diagnosis, prognosis and treatment.^[4,5]^ Most of the current research is focused on lncRNA and MicroRNA (miRNA), and there is currently insufficient understanding of the interaction between lncRNA, miRNA and mRNA (messenger RNA). There are some reports that the mechanisms of lncRNA, miRNA and mRNA in the development of cancer and their interactions.^[6–8]^ The miRNA-mRNA-lncRNA network provides clues for understanding the mechanism of tumors and developing novel diagnostic and therapeutic strategies.

The long noncoding RNAs (lncRNAs, >200 nucleotides in length) are increasingly becoming a research hotspot. Studies have pointed out that the lncRNA DLX6-AS1 can promote cell growth and invasiveness in bladder cancer.^[9]^ There are also studies that point out that the lncRNA TC0101441 can induces epithelial-mesenchymal transition in epithelial ovarian cancer metastasis.^[10]^ So the lncRNAs may be involved in many biological processes that regulate tumorigenesis and cancer metastasis. There are also reports that there is a relationship between the lncRNAs polymorphisms and the risk of cancer. Research by Moschovis et al suggested that polymorphisms of these 2 lncRNAs polymorphisms (PVT1 rs1561927 and HOTAIR rs4759313) implicated in pancreatic carcinogenesis.^[6]^ Yuan et al prove that single nucleotide polymorphism rs114020893 of NEXN-AS1 at 1p31.1 may contribute to lung cancer susceptibility.^[12]^

We found a lot of studies on LINC00673 rs11655237 C>T polymorphism and tumor susceptibility, however, there is some controversy about its relationship with tumor susceptibility. Therefore, we performed this meta-analysis to more comprehensively and precisely assess the association between the LINC00673 rs11655237 C> T polymorphism and cancer risk.

## 2. Materials and Methods

### 2.1. Search strategy

A Medical Subject Headings method was used to retrieve subject terms from databases. “LINC00673” or “SLNCR1” and “rs11655237” and “polymorphism” or “mutation” or “variant” and “cancer” or “carcinoma” or “tumor” or “neoplasms” were used as keywords. The keywords were used as searching terms for PubMed, the Cochrane Library, Embase databases, Web of Science, China National Knowledge Infrastructure, and Wanfang Databases. Studies published up to October 20, 2019 were included. Selected articles were not confined to any language publications.

### 2.2. Inclusion and exclusion criteria

The articles included in this study must meet the following criteria:

1. independently published cohort design or case–control studies.
2. sufficient information for calculating the odds ratios (ORs) with its 95% confidence intervals (CIs).
3. The relationship between the LINC00673 rs11655237 polymorphism and cancer is evaluated in the study.
4. Hardy-Weinberg equilibrium (HWE).

The exclusion criteria were as follows:

1. reviews, letters, editorials, and case reports.
2. genotype frequency was not clear.
3. family-based studies.
4. not in HWE.

### 2.3. Data extraction

Two researchers (Liang and Ke) independently evaluated and extracted information from eligible studies. The data extracted included the following information: the first author’s name, year of publication, the source of specimens of cases; genotyping method, cancer type, total number of cases and controls, genotype of the cases and controls, HWE was calculated from the study data. If the two investigators have any different opinions, submit the dispute to the third investigator (Zhang) until a consensus was reached.

### 2.4. Quality score assessment

The Newcastle-Ottawa Scale Quality Assessment Scale was used to assess the methodological quality (http://www.ohri.ca/programs/clinical_epidemiology/nosgen.Pdf). This scale is based on three aspects: selection of the study sample (4 items, 4 points); comparability of the sample groups (2 items, 2 points) and; ascertainment of exposure/ outcome (3 items, 3 points). The higher the score, the better the quality of the document, the scores range from 0 (worst) to 9 (best) score. the highest is 9 points; the definition of ≥6 points for high quality literature.^[13]^

### 2.5. Statistical analysis

We evaluated the association between the LINC00673 rs11655237 polymorphism and risk of cancer by using different comparison models: allelic model (T vs C), homozygous model (TT vs CC), heterozygous model (CT vs CC), recessive model (TT vs CT+ CC), and dominant model (CT + TT vs CC). HWE in the controls of each study was evaluated with the Goodness-of-fit Chi-squared test, and *P* < .05 was indicated the study departed from HWE. The ORs and the corresponding 95% CIs were calculated according to the frequencies of genotypes. Heterogeneity of these studies was assessed with a chi-square *Q* test and *I*^2^ statistics. If no significant heterogeneity was observed with *P*-value <.1 or *I*^2^ > 50 %, we considered the heterogeneity significant, a random-effects model (DerSimonian and Laird method) was adopted. Otherwise, the fixed-effects model (the Mantel-Haenszel method) was applied. We also performed a subgroup analysis based on cancer type. Sensitivity analyses were performed to examine possible changes when single studies were excluded. Moreover, we used Begg’s funnel plot and Egger’s test to investigate the potential publication bias in the meta-analysis.

All the statistical analyses in this meta-analysis were conducted with STATA software (version 12.0; Stata Corporation, College Station, Texas, USA). All the *P*-values were 2-sided, and *P* < .05 was considered that the result was statistically significant.

## 3. Results

### 3.1. Literature search

**Figure 1.**
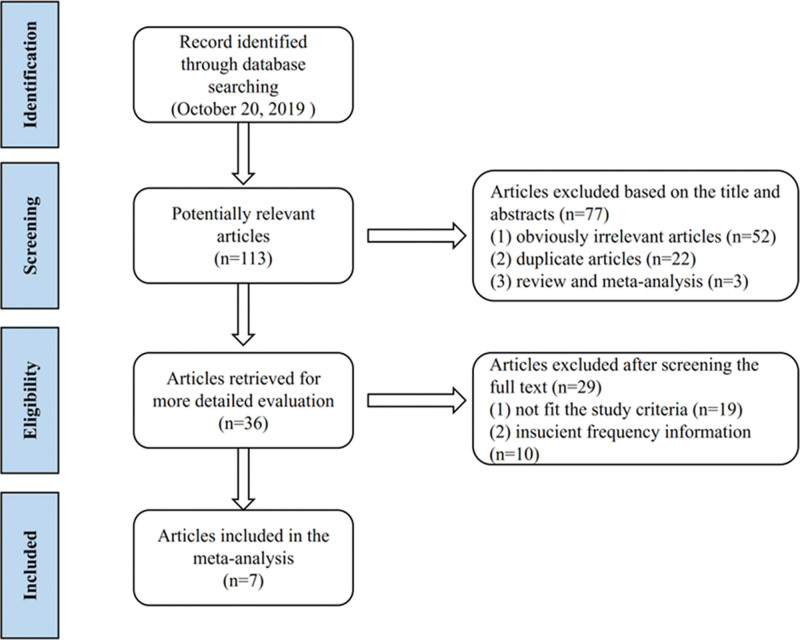
Flow diagram of literature selection.

After searching the database based on search terms, 113 related studies were retrieved, including 74 from PubMed, 13 from EMBASE, 1 from Cochrane Library, 24 from Web of Science, 0 from China National Knowledge Infrastructure, and 1 from Wanfang Databases. First, delete duplicate records, obviously irrelevant articles, duplicate articles and review and meta-analysis, of which 77 studies were deleted. Of the remaining 36 studies, the 29 papers were excluded after reading the full text, 19 were not in compliance with the research criteria, and 10 were insufficient in frequency information. Finally, 7 articles including 7 case-control studies, 7423 cases and 11,049 controls were identified as suitable for meta-analysis.^[14–20]^ The flow of the study is shown in Figure 1.

### 3.2. Main characteristics of included studies

Seven case-control studies were published between 2014 and 2019, and they were all conducted in China, these studies are all about Asian studies. The tumor types included in the study were Wilms tumor, hepatoblastoma, gastric cancer (GC), neuroblastoma, cervical cancer, pancreatic cancer. The genotype frequencies of both case and control groups were obtained from the original study. The source of the control population was all hospital-based. HWE was calculated from the genotype of the control population, and all studies are consistent with HWE. The Newcastle-Ottawa Scale quality score shows that all 7 studies were all “high quality.” All the studies applied TaqMan probe technology (TaqMan). The characteristics of the included studies are shown in Table 1.

**Table 1:** Characteristics of the studies included in the meta-analysis and their genotype distributions of the LINC00673 rs11655237 polymorphism.

					Genotyping			Cases			Controls				
Author	Year	Country	Design	Cancer type	Method	Cases	Controls	CC	CT	TT	CC	CT	TT	HWE	NOS
Gao	2018	China	HB	Wilms tumor	TaqMan	145	531	92	48	5	325	178	28	0.575	7
Yang	2019	China	HB	Hepatoblastoma	TaqMan	213	958	115	85	13	595	312	51	0.232	6
Zhao	2019	China	HB	Gastric cancer	TaqMan	1392	1364	775	522	95	838	458	68	0.596	7
Li	2019	China	HB	Neuroblastoma	TaqMan	698	1516	401	252	45	935	513	68	0.824	8
Zhang	2018	China	HB	Neuroblastoma	TaqMan	391	812	218	146	27	505	272	35	0.831	7
Wang	2018	China	HB	Cervical cancer	TaqMan	1000	1000	561	374	65	615	338	47	0.949	8
Zheng	2014	China	HB	Pancreatic cancer	TaqMan	3584	4868	1961	1355	268	2990	1623	255	0.157	9

HB = hospital based, HWE = Hardy-Weinberg equilibrium, NOS = Newcastle-Ottawa Scale, TaqMan = TaqMan probe technology.

### 3.3. Meta-analysis results

Heterogeneity has not been found in these seven studies for all the 5 genetic models. Therefore, we used fixed effect model to calculate the ORs and their 95% CIs. Association between LINC00673 rs11655237 gene polymorphisms and cancer risk was shown in Table 1 under all the 5 genetic models: allelic model (T vs C): OR = 1.24, 95% CI = 1.18 to 1.31, *P* < .001; homozygous model (TT vs CC): OR = 1.54, 95% CI = 1.36 to 1.76, *P* < .001; heterozygous model (CT vs CC): OR = 1.24, 95% CI = 1.16 to 1.32, *P* < .001; dominant model (CT + TT vs CC): OR = 1.28, 95% CI = 1.20 to 1.36, *P* < .001; recessive model (TT vs CT+ CC): OR = 1.42, 95% CI = 1.25 to 1.61, *P* < .001. A significant association between LINC00673 rs11655237 gene polymorphisms and cancer risk was observed under all the 5 genetic models (Table 2). The LINC00673 rs11655237 C>T polymorphism may be associated with cancer susceptibility.

**Table 2:** Summary of the association between LINC00673 rs11655237 polymorphism and cancer risk.

Genetic model	Subgroup	*P*_*H*_	Effect model	OR (95% CI)	*P*_Z_
Allelic model	Overall	.496	Fixed	1.24 (1.18–1.31)	<.001
(T vs C)	Others	.294	Fixed	1.25 (1.18–1.32)	<.001
	Neuroblastoma	.540	Fixed	1.23 (1.09–1.38)	.001
Homozygous model	Overall	.687	Fixed	1.54 (1.36–1.76)	<.001
(TT vs CC)	Others	.461	Fixed	1.53 (1.33–1.76)	<.001
	Neuroblastoma	.662	Fixed	1.63 (1.19–2.23)	.003
Heterozygous model	Overall	.757	Fixed	1.24 (1.16–1.32)	<.001
(CT vs CC)	Others	.613	Fixed	1.25 (1.17–1.34)	<.001
	Neuroblastoma	.614	Fixed	1.18 (1.01–1.37)	.035
Dominant model	Overall	.602	Fixed	1.28 (1.20–1.36)	<.001
(CT + TT vs CC)	Others	.417	Fixed	1.29 (1.20–1.38)	<.001
	Neuroblastoma	.559	Fixed	1.23 (1.06–1.36)	.005
Recessive model	Overall	.751	Fixed	1.42 (1.25–1.61)	<.001
(TT vs CT+ CC)	Others	.545	Fixed	1.40 (1.22–1.61)	<.001
	Neuroblastoma	.727	Fixed	1.53 (1.12–2.09)	.007

CIs = confidence intervals, ORs = odds ratios.

**Figure 2.**
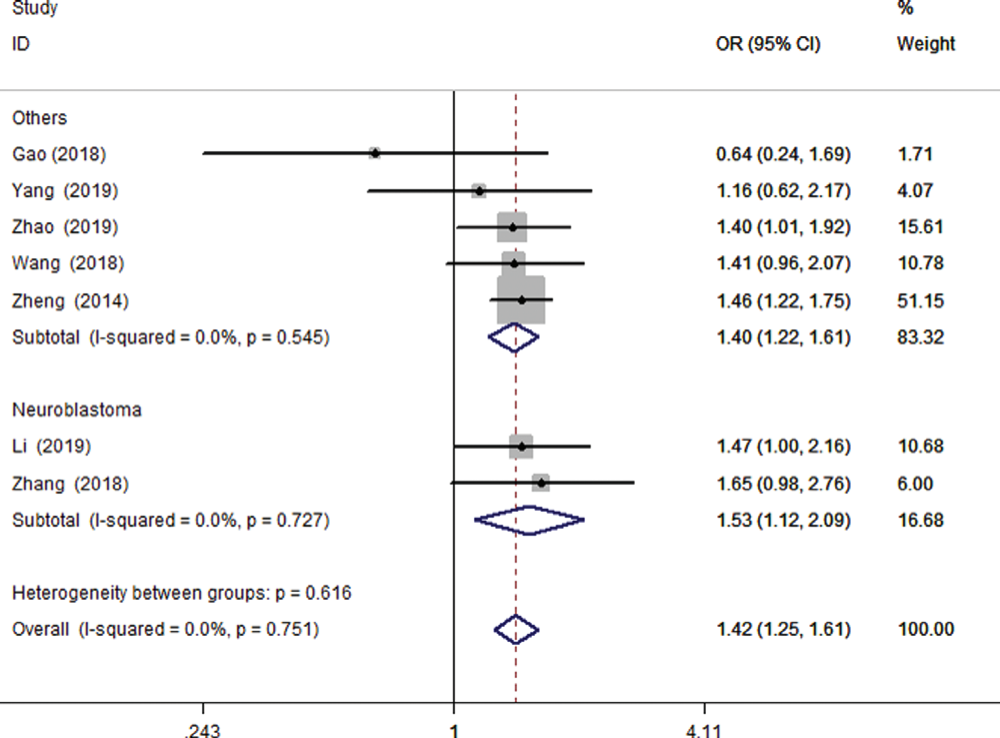
Forest plot of cancer risk associated with LINC00673 rs11655237 polymorphism in the overall analysis (Recessive model TT vs CT+ CC).

Because the control groups included in the study were based on hospital specimens, all were published by Chinese and the DNA typing techniques used were TaqMan, and they are all high-quality research. In addition, the type of tumor is not exactly the same, two of which are related to neuroblastoma, so the subgroup analysis is mainly divided into two groups: neuroblastoma and others (Wilms tumor, hepatoblastoma, GC, cervical cancer, pancreatic cancer). Subgroup analysis based on tumor type, we found no significant changes in heterogeneity under all genetic models. Subgroup analysis also shows that a significant association between the LINC00673 rs11655237 gene polymorphism and neuroblastoma risk. Forest plot shows the association between LINC00673 rs11655237 polymorphism and cancer risk in Figure 2 (Recessive model: TT vs CT+ CC).

### 3.4. Sensitivity analysis and publication bias

**Figure 3.**
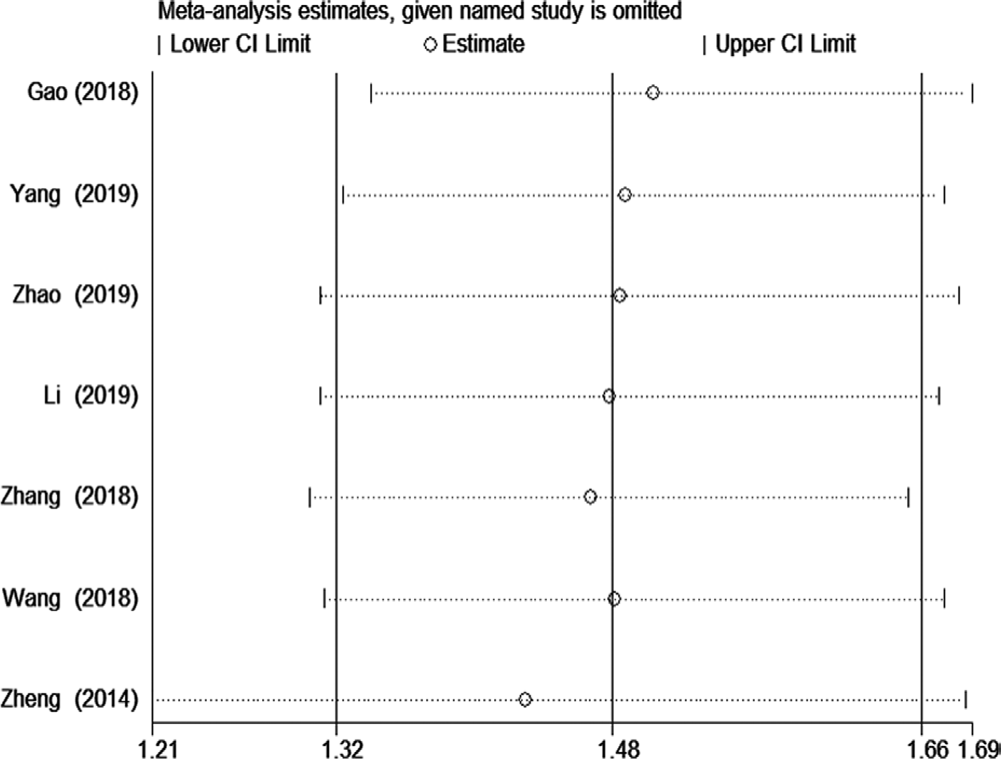
Sensitivity analysis between LINC00673 rs11655237 polymorphism and cancer risk (homozygous model TT vs CC).

**Figure 4.**
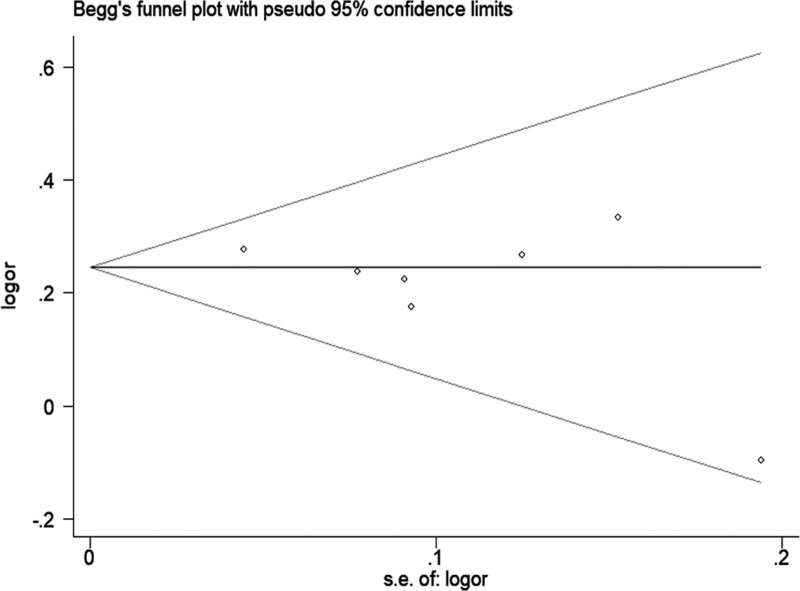
Publication bias between LINC00673 rs11655237 polymorphism and cancer risk (dominant model CT + TT vs CC).

The sensitivity analysis was performed to evaluate the influence of each study on the merged OR. Finally, there is no qualitative change in the corresponding merged ORs. This reinforces the certainty and reliability of the results. The sensitivity analysis between LINC00673 rs11655237 polymorphism and cancer risk is shown in Figure 3 (homozygous model: TT vs CC). Funnel plots were used to investigate publication bias in the meta-analysis. We observed a relatively symmetric distribution in the funnel plot in the all models, which indicates that there is no significant publication bias in the included studies. The *P* value of the Egger’s test are >0.05 in all models. The publication bias of the dominant model (CT + TT vs CC) is shown in Figure 4.

## 4. Discussion

The progression of cancer is a complex process that is the result of interactions between human genetic factors and external surroundings factors. Physical carcinogens (such as ultraviolet), chemical carcinogens (such as aflatoxin), and biological carcinogens (such as infections from certain viruses, bacteria) are external factors in cancer risk. The genetic factors of cancer risk are mainly the relationship between gene polymorphism and tumor susceptibility, and the interaction between genes and the environment. The causes of cancer’s occurrence and development are not fully understood. But genetic polymorphism may explain it in the field of genetics.

LINC00673 is located at 17q24.3, which exhibits a high frequency of loss of heterozygosity. The relationship between LINC00673 rs11655237C> T polymorphism and cancer susceptibility has been demonstrated. Zhang et al pointed out that the rs11655237 T allele was positively associated with neuroblastoma and could confer neuroblastoma susceptibility (OR =1.80, 95% CI = 1.06–3.06, *P* = .029).^[18]^ The results of LI et al showed that the polymorphism of LINC00673 rs11655237 C> T increased the risk of neuroblastoma in Chinese children (TT vs. CC: OR = 1.58, 95% CI = 1.06–2.35, *P* = .024; additive model: OR = 1.20, 95% CI = 1.03–1.39, *P* = .020; recessive model: OR = 1.50, 95% CI = 1.02–2.22, *P* = .040).^[15]^ Zhao et al found that rs11655237 polymorphism significantly increased GC risk in the Chinese population (OR = 1.29, 95% CI = 1.12–1.48, *P* = 4.1 × 10^-4^), and a significant interaction was found between rs11655237 and Helicobacter pylori infection (*P* = .006).^[19]^ Yang et al found that the LINC00673 rs11655237 C> T polymorphism can increase the risk of hepatoblastoma (CT+TT vs CC: OR = 1.40, 95% CI = 1.04–1.88, *P* = .029).^[17]^ Gao et al showed that LINC00673 rs11655237 C> T polymorphism was not significantly associated with Wilms tumor susceptibility under all the tested genetic models (CT vs CC: OR = 0.94, 95% CI = 0.63–1.40; TT vs CC: OR = 0.60, 95% CI = 0.22–1.59; TT/CT vs CC: OR = 0.89, 95% CI = 0.61–1.31; and TT vs CC/CT: OR = 0.61, 95% CI = 0.23–1.61).^[14]^ Wang et al found rs11655237 significantly increased susceptibility of cervical cancer in a Chinese population (OR = 1.27, 95% CI = 1.08–1.50; *P* = .005).^[16]^ Zheng et al pointed out that LINC00673 rs11655237C>T is associated with pancreatic cancer susceptibility and indicates that, CT genotype carriers have a 28% increased risk of pancreatic cancer compared with CC genotype (OR = 1.28, 95% CI = 1.16–1.41, *P* = .121).^[20]^ We found that Gao’s research conclusions are inconsistent with other people’s research conclusions. There may be two reasons, first, the sample size is too small; second, selection bias and information bias are inevitable in their retrospective study. We conducted a meta-analysis of the above literature and found that the LINC00673 rs11655237C>T polymorphism can increase the risk of cancer (allelic model T vs C: pooled OR = 1.24, 95% CI = 1.16–1.41, *P* < .001; homozygous model TT vs CC: pooled OR = 1.54, 95% CI = 1.36–1.76, *P* < .001; heterozygous model CT vs CC: pooled OR = 1.24, 95% CI = 1.16–1.32, *P* < .001; dominant model CT + TT vs CC: pooled OR = 1.28, 95% CI = 1.20–1.36, *P* < .001; recessive model TT vs CT+ CC: pooled OR = 1.42, 95% CI = 1.25–1.61, *P* < .001). Subgroup analysis also demonstrated that polymorphisms at this site also increased the risk of neuroblastoma.

LncRNAs play a crucial role in the development and progression of tumors. It increases tumor risk and may be related to the following signaling pathways: Firstly, LINC00673 was found to regulate MARK4 expression by mutagenizing miR-515-5p, thereby inhibiting the Hippo signaling pathway, finally, the transcription factor Yin Yang 1 binds to the LINC00673 promoter and increases its cis transcription.^[21]^ Secondly, LINC00673 is able to reinforce the interaction of PTPN11 with PRPF19, an E3 ubiquitin ligase, and promote PTPN11 degradation through ubiquitination, which causes diminished SRC-ERK oncogenic signaling and enhanced activation of the STAT1-dependent antitumor response.^[22]^ In addition LINC00673-v4 augmented the interaction between DDX3 and CK1ε and thus the phosphorylation of dishevelled, which subsequently activated WNT/β-catenin signaling and consequently caused aggressiveness of cancer.^[23]^ Of course, lncRNAs can also regulate the invasion, proliferation, and metastasis of tumor cells through other pathways.^[24–26]^ All of the above studies provide reliable evidence that lncRNALINC00673 plays an important role in the development, progression, and metastasis of various cancers.

However, there are some limitations that exist in our meta-analysis. In our meta-analysis, only the relationship between the polymorphism of LINC00673 rs11655237 C> T and the susceptibility of tumor was studied. Other sites of the gene and the interaction of genes with the external environment were not analyzed. Small sample size may be the biggest research defect, which may affect the result and lead to incorrect conclusions. The subjects included in the study are all Chinese, so racial bias is inevitable and affects the outcome.

In conclusion, our meta-analysis showed that the LINC00673 rs11655237 C> T polymorphism can increase cancer susceptibility. Given the current limitations of this meta-analysis, a larger sample size for further research is needed in the future and multiple ethnic participants should participate to clarify the association between LINC00673 rs11655237 C> T polymorphism and cancer risk.

## Author contributions

**Conceptualization:** Xingxuan Ma.

**Data curation:** Hongyu Zhang, You He.

**Formal analysis:** Xingxuan Ma.

**Investigation:** Liuhua Ke.

**Methodology:** Ka Liang.

**Project administration:** Ka Liang.

**Software:** Baixiu Wu, Ka Liang, Liuhua Ke, Xingxuan Ma, Changliu Luo.

**Supervision:** Liuhua Ke.

**Visualization:** Liuhua Ke.
